# Variations in power of opinion leaders in online communication networks

**DOI:** 10.1098/rsos.180642

**Published:** 2018-10-17

**Authors:** Mohsin Adalat, Muaz A. Niazi, Athanasios V. Vasilakos

**Affiliations:** 1 COSMOSE Research Group, Department of Computer Science, COMSATS University Islamabad, Islamabad, Pakistan; 2 Department of Computer Science, Lulea University of Technology, Lulea, Sweden

**Keywords:** complex networks, social network analysis, Panama papers, opinion formation, complex adaptive system, Panama leaks

## Abstract

Online social media has completely transformed how we communicate with each other. While online discussion platforms are available in the form of applications and websites, an emergent outcome of this transformation is the phenomenon of ‘opinion leaders’. A number of previous studies have been presented to identify opinion leaders in online discussion networks. In particular, Feng (2016 *Comput. Hum. Behav.* **54**, 43–53. (doi:10.1016/j.chb.2015.07.052)) has identified five different types of central users besides outlining their communication patterns in an online communication network. However, the presented work focuses on a limited time span. The question remains as to whether similar communication patterns exist that will stand the test of time over longer periods. Here, we present a critical analysis of the Feng framework both for short-term as well as for longer periods. Additionally, for validation, we take another case study presented by Udanor *et al.* (2016 *Program* **50**, 481–507. (doi:10.1108/PROG-02-2016-0011)) to further understand these dynamics. Results indicate that not all Feng-based central users may be identifiable in the longer term. Conversation starter and influencers were noted as opinion leaders in the network. These users play an important role as information sources in long-term discussions. Whereas network builder and active engager help in connecting otherwise sparse communities. Furthermore, we discuss the changing positions of opinion leaders and their power to keep isolates interested in an online discussion network.

## 1. Introduction

Owing to the advancements in online social networks, our modes of communication have been undergoing a massive transformation. Nowadays, people employ the use of online discussions more so than face-to-face discussions. While exact platforms keep coming and going, some of the currently active social media platforms include Facebook, Instagram and Google+ among others [1]. These platforms not only connect users with their friends and family but also give them the opportunity to stay abreast of news and events. Rapid information diffusion in the large online community is one of the key features of online social networks popularity.

Cherepnalkoski *et al.* have presented a recent study noting Twitter to be one of the most popular online microblogging social networking platforms with over 300 million active users [2]. Twitter enables users not only to create content but also to share opinions with a massive online global community [3]. Users often include celebrities and politicians using Twitter as a channel of communication to promote their personal profile as well as opinions. This heterogeneous online communication between large numbers of users generates a tremendous amount of large-scale unstructured data. This large-scale unstructured data can be used to identify trends, influential users [4], communication patterns and opinion/behaviour of participants [5] involved in online communication.

One particular way of analysing online social networks is to convert them to graphs [6]. Once converted, Social network analysis can be employed as a tool for the exploration and analysis of large-scale unstructured data [7]. Social network models can vary significantly including applications as varied as biomedical research networks [8], vehicular social networks [9] and biological networks [10]. However, previous work has noted the variations in measurement of intelligence used for analysing complex data [11]. Previously, a number of studies have been carried out using large-scale Twitter data in different fields like human behaviours [12,13], medical [14], opinion formation [15], link prediction [16] and many others. Ch’ng used Twitter data to analyse formation of online communities [17] and Baek *et al.* argued roles and positions of participants in an online community [18]. Geo-located tweets are used by Blanford *et al.* to capture regional connections and cross-border movement [19]. Twitter data are even used for evaluation of inter-organizational disaster coordination networks [20], created in the result of disaster. Lee *et al.* demonstrated how scholars are using the Twitter communication network for informal scholarly communication [21]. The aim of all the above studies in different fields is to find the influential nodes in the network. In an online communication network, these nodes are called opinion leaders.

Rogers defined opinion leadership [22] as ‘the degree to which an individual is able to influence other individuals’ attitudes or overt behaviour informally in desired way with relative frequency’. The concept of opinion leadership originates from two-step flow theory. The theory posits that information first comes from mass media to opinion leaders and then this information is transmitted to society via opinion leaders [23]. Opinion leaders are opinion brokers in two senses: first, opinion leaders' influence is between two social groups rather than in-between social group, and second, they are transition between two network mechanism responsible for spreading the idea [24].

Regarding opinion leaders' identification, many studies have been carried out [25–29]. Feng proposed a novel approach for the identification of most central users in an online communication network [30] by adopting two-step flow theory [23]. The author identified five types of central users in the Twitter network: *conversation starter*, *influencer*, *active engager*, *network builder* and *information bridge*. Feng analysed tweets in only 4 days time span with the goal to trace communication patterns. However, opinion in a society may vary with time. Thus, it is clear that proposed approach needs to be applied for trend analysis on a longitudinal network.

For this purpose, we use #PanamaLeaks as a case study since it is one of the most long-debated Pakistani hashtags discussed on the Twitter network. International Consortium of Investigative Journalists raised the issue of Panama Leaks by leaking 11.1 million records from the Panama-based law firm, Mossack Fonseca [31]. The leaked documents contain information about offshore investments of many wealthy individuals including political leaders of many countries. Panama leaks became popular in Pakistan, as former Prime Minister of Pakistan was one of the many Pakistani nationals mentioned in this leak. The political parties of Pakistan filed a case against former Prime Minister of Pakistan in Supreme Court of Pakistan based on this leak. In response #PanamaLeaks became the most popular Twitter trend in Pakistan. Our long-term analysis shows that very few people are influencing this online discussion. We also identify types of these influential participants in the long-term online discussion as well as the impacts of these central participants over the time.

### 1.1. Motivation

This study is motivated by the lack of study for the identification of different types of central users and their impact on communication in online communication networks in a long-term manner. The studies mentioned in the above section focus on the identification of opinion leaders but fail to identify the types of the most important participants involved in online discussion and their impacts on the online communication over time. In this context, this study critically analyses the previous approach proposed by Feng [30]. We explore communication patterns among participants of an online communication network in short-term and long-term manner. We use two case studies, ‘PanamaLeaks’ and ‘NigeriaDecides’. We use short-term analysis to validate our work by using previously published dataset used in [32] as previous work by Feng also used tweets of a limited time span. However, opinion in a society varies with the time. Therefore, we explore the patterns between central users of an online communication network in a long-term manner by using ‘PanamaLeaks’ as a case study. Furthermore, we are interested in the roles and positions of the central users in an online discussion network over the time. We have also addressed the impact of these central users on the isolates participating in the online discussion. Researchers have claimed that the Internet enables individuals to influence others by using their social network connections and by replicating their offline asymmetric power of influence into the online social networking platforms [33,34]. In this context, this study also explores the flow of information in the online discussion network of #PanamaLeaks in order to understand how very few people have prevailed in the online discussion. Bringing together these concerns our research objective is as follows:

RO: Analysing the interaction patterns of central users to evaluate the influence in long-term online social media networks.

### 1.2. Main contributions

Key contributions of this study are as follows:

— We critically analyse the framework presented by Feng in a long-term manner. We show that not all of the central users identified by the Feng study may exist in an online communication network in a long-term manner. For further analysis as well as for validation, we use another previously published case study.
— We reveal the variation in the power of opinion leaders in a long-term online communication network. We show that the power of opinion leaders to influence others in the online communication may not remain constant with the passage of time.
— Our work demonstrates the impact of central users on the duration of a trend in an online communication network. Results show that isolates are only interested in the online discussion while central users keep their interest in the trend.

## 2. Central users in online communication network

In this section, we present five types of central users identified by Feng in online communication network [30].

### 2.1. Conversation starter user

**Figure 1.**
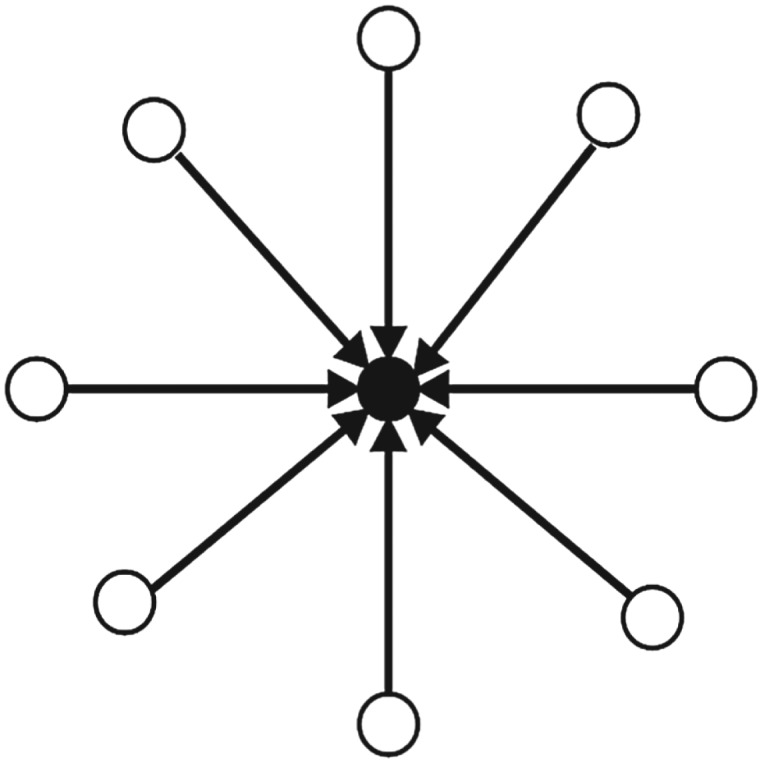
Connection Pattern for ‘Conversation Starter’ in an online discussion network. Circles are presenting users in the network. A circle in the middle filled with black colour is ‘Conversation Starter’. Many arrows pointing towards ‘Conversation Starter’ presents many users mentioning/retweeting ‘Conversation Starter’ tweets in the online discussion network.

A *conversation starter* is a user in a network with numerous ‘in-degree’ links and a few or none ‘out-degree’ links. *Conversation starter* is the one who is responsible for starting the original topic and the flow of information in the network. However, the control on the flow of information in the online communication network is not under control of the *conversation starter*. Figure 1 shows the connection pattern for *conversation starter* in the network.

### 2.2. Influencer user

**Figure 2.**
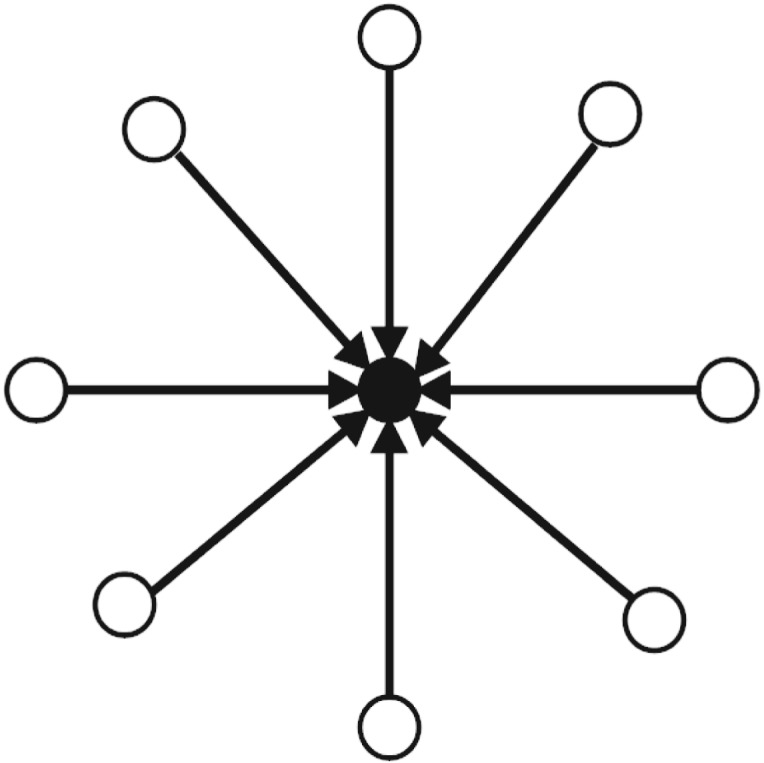
Connection Pattern for ‘Influencer’ in an online discussion network. Circles are presenting users in the network. A circle in the middle filled with black colour is ‘Influencer’. Many arrows pointing towards ‘Influencer’ presents many users mentioning/retweeting ‘Influencer's’ tweets in the online discussion network.

An *influencer* is opinion leader in the network. *Influencer* in a network has plentiful ‘in-degree’ links and few ‘out-degree’ links. *Influencer* does not initiate the topic of conversation in the network; however, *influencer* does influence opinion of other users in the network by creating frequent tweets that are retweeted by many isolates. Figure 2 shows the connection pattern for *influencer* in the network.

### 2.3. Active engager user

**Figure 3.**
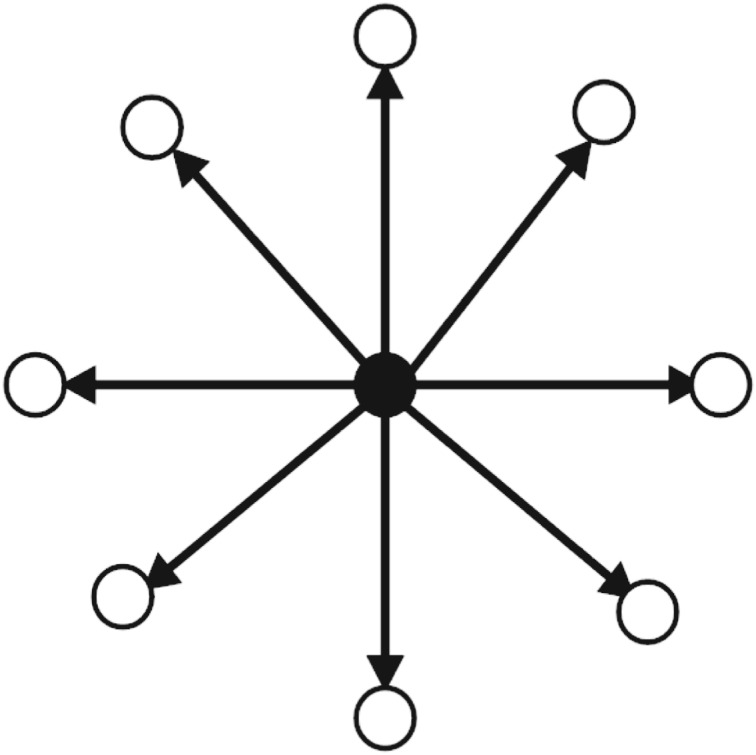
Connection Pattern for ‘Active Engager’ in an online discussion network. Circles are presenting users in the network. A circle in the middle filled with black colour is ‘Active Engager’. Many arrows pointing towards users from ‘Active Engager’ presents that ‘Active Engager’ mentions/retweets many other users' tweets in the online discussion network.

An *active engager* is a user in online discussion network with many ‘out-degree’ and a few or none ‘in-degree’ links. *Active engager* is eager to distribute information and build connections with other users in an online discussion network. *Active engager* is also opinion expresser in network. Figure 3 shows the connection pattern for *active engager* in the network.

### 2.4. Network builder user

**Figure 4.**
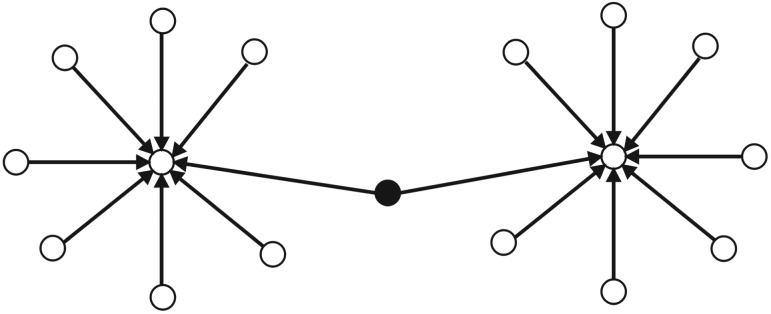
Connection Pattern for ‘Network Builder’ in an online discussion network. Circles are presenting users in the network. A circle in the middle filled with black colour is ‘Network Builder’. Two arrows pointing towards ‘Influencers’ from the ‘Network Builder’ describe the primary role of ‘Network Builder’ in the network.

Despite having a few ‘out-degree’ and a few or none ‘in-degree’ links in an online discussion network, *network builder* plays an important role in the online discussion. The primary role of *network builder* is connecting two or more *influencers* in the network. Figure 4 shows the connection pattern for *network builder* in the network.

### 2.5. Information bridge user

**Figure 5.**
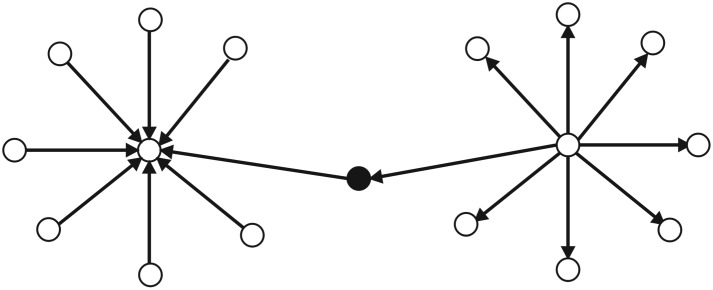
Connection Pattern for ‘Information Bridge’ in an online discussion network. Circles are presenting users in the network. A circle in the middle filled with black colour is ‘Information Bridge’. Arrow pointing towards ‘Influencer’ in the network is presenting that ‘Information Bridge’ has mentioned/retweeted ‘Influencer’ tweet and arrow from ‘Active Engager’ towards ‘Information Bridge’ is presenting that same tweet is retweeted by the ‘Active Engager’.

An *information bridge* is a user in online discussion network with a few ‘in-degree’ and ‘out-degree’ links. *Information bridge* role is to assist *influencer* and *active engager* in the network to connect with other users [30]. Note that in both case studies, #PanamaLeaks and #NigeriaDecides which we took for validation, we are unable to identify such user in the online discussion networks (see §5). Figure 5 shows the connection pattern for *information bridge* user identified by Feng in the online discussion network.

## 3. Proposed methodology

### 3.1. Tools used

We chose NodeXL and Gephi as tools to analyse #PanamaLeaks network. NodeXL is an extensible toolkit to download and analyse network data [35]. NodeXL is capable of calculating network matrices like ‘in-degree’, ‘out-degree’, ‘betweenness’, ‘density’, ‘modularity’, etc., NodeXL generates Excel-based reports, and it is also capable of downloading and analysing data from several social network platforms like Twitter, Flickr and Facebook.

In a Twitter user network, a Twitter user is presented as a vertex in network generated by NodeXL and edge represents the relationship between two users. The relationship between two vertices can be direct or undirected. In a Twitter user network, ‘in-degree’ of each vertex is how many users in the online discussion network have mentioned or retweeted his/her tweets and ‘out-degree’ of a vertex is how many times he/she has mentioned/retweeted other participants in his/her tweets. NodeXL adds an edge when one user tweets, retweets or mentions other user/users in his/her tweet. Edges coming towards one user are ‘in-degree’ of that specific user and edges going from that user to others in the network are ‘out-degree’ of that specific user. If a user creates new tweet/tweets without mentioning, replying or retweeting another user’s tweet, NodeXL adds self-loop around that user in the network.

Along with finding the most central users of #PanamaLeaks network, we aim to visualize #PanamaLeaks network dynamically to find structural changes in the network. We chose Gephi, a social network analysis tool [36], for network visualization, due to its capability for visualizing large, complex and dynamic networks. Gephi is capable of visualizing network with many different algorithms like the Force-Atlas, Fruchterman–Reingold, Yifan Hu, etc. [37].

### 3.2. Dataset

We used NodeXL Twitter Search Network data collector to collect tweets from March 2017 to August 2017. We chose #PanamaLeaks as a keyword to download tweets. Our dataset contains 10 612 nodes and 24 623 unique edges. Although we used Twitter Slandered Search API using NodeXL to download tweets, this Twitter API has some limitations, like 18 000 tweets per day and no assurance of getting complete data regarding that particular hashtag.

For further analysis and validation, we used #NigeriaDecides case study dataset [32]. The author used #NigeriaDecides as a query to download tweets and then generate a network of tweets, mentions and replies, which is same as #PanamaLeaks case study network. #NigeriaDecides case study dataset consists of 1752 nodes and 6343 edges from 15 April 2015 to 20 April 2015.

### 3.3. Methods

We combine all the data (tweets downloaded using NodeXL during six months) and then divide it into six months of different networks according to the dates of tweets from March 2017 to August 2017. We requested NodeXL to generate the following matrices: ‘in-degree’, ‘out-degree’, ‘betweenness centrality’, ‘density’, ‘clustering coefficient’ and ‘modularity’ for each month independently. ‘In-degree’ is a simple count of unique incoming connections/links towards a user/entity in a directed graph. Whereas ‘out-degree’ is a simple count of unique links/connections from a user. ‘Betweenness centrality’ of the node (a unique user in a network) is how many times a node appears in the shortest path between other nodes (other users in the same network) [38].

‘In-degree’, ‘out-degree’ and ‘betweenness centrality’ are the three measures used by Feng [30] for identification of most central users in an online discussion network. This is the first reason we also used these three measures on the #PanamaLeaks case study. Furthermore, degree centrality is the only measure through which we can find the number of participants replying, mentioning and retweeting tweets of other participants in the online discussion network. This indicates the influence of a participant in the online social network [18]. Some researchers have also used ‘in-degree’ to identify hubs in the online discussion network [39]. Empirical results show that degree centrality is the most effective indicator of the structural importance of a participant in the social network [40]. Moreover, the node with the high ‘betweenness centrality’ has the high power of diffusion in the network [41]. Participants with high ‘betweenness centrality’ in the social network can influence the flow of information and they have the ability to connect otherwise sparse communities [42]. It is also an effective indicator of a user lead role in the online social network [43] and shows the access of a participant to the novel information in the network [44]. Empirical results show that not only in social networks but also in a biological network important proteins have high ‘betweenness centrality’ [45]. In this work, our focus is the core of resources where the conversation in online communication takes place and the power to influence the flow of information in the online discussions. It seems like in both the above cases ‘betweenness centrality’ is an effective measure to use [46,47].

We identified top 10 central users based on ‘betweenness centrality’ for each month in the #PanamaLeaks network. We adopted the same methods in the #NigeriaDecides case study used in [32] for validation purpose. For visualization of connection patterns for most central users in #PanamaLeaks network, we use Yifan Hu algorithm for both case studies. Note: we avoid using full user names due to privacy concerns.

Figure 7 shows the connection pattern for a *conversation starter* for #PanamaLeaks and #NigeriaDecides. Figures 8, 10 and 12 show connection patterns for *influencer*, *active engager* and *network builder* in the #PanamaLeaks network for each month from April 2017 to August 2017. Figures 9, 11 and 13 show the connection patterns for central users of #NigeriaDecides network.

We used social network analysis measures like ‘density’, ‘clustering coefficient’, and ‘modularity’ to analyse overall communication patterns among the participants of the #PanamaLeaks network. To check the impact of *conversation starter* and *influencer* on the online discussion network, we use number of unique users coming into #PanamaLeaks network every month.

**Figure 6.**
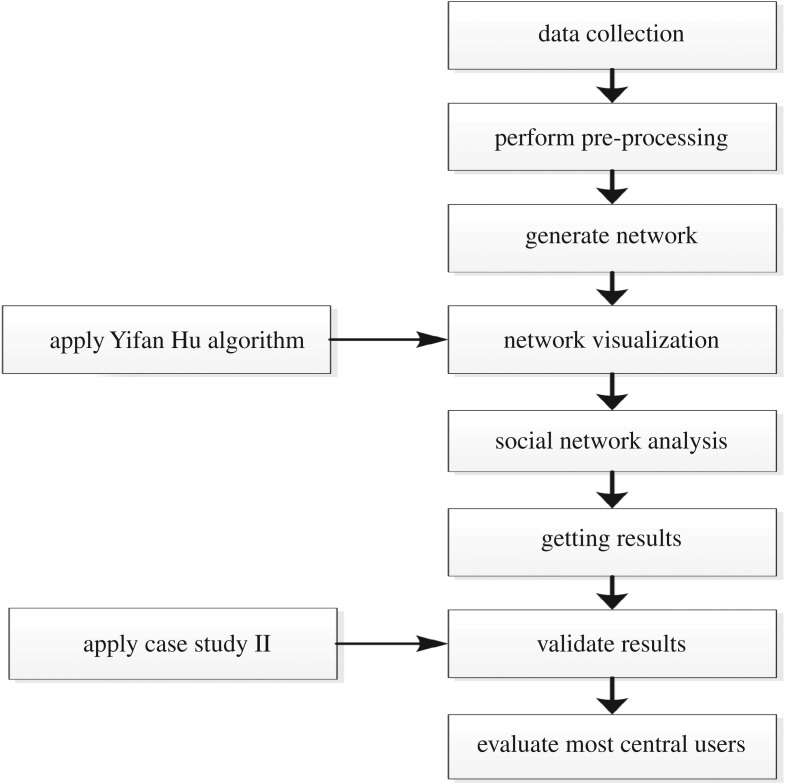
Research method. Our work starts with data collection step. The collected data were organized by performing the pre-processing technique. Networks were generated from well-organized data. Then, we applied the Yifan Hu algorithm on the network for visualization purpose. The next step was performing social network analysis and getting results. For further analysis as well as validation, the same approach was applied on another case study of #NigeriaDecides and then we have evaluated most central users in the network.

For visualization of overall communication pattern among #PanamaLeaks network participants, we created a dynamic network of #PanamaLeaks using Gephi. We implemented month-wise timestamp on the network based on tweet date. We used Yifan Hu algorithm, one of the many available algorithms for network visualization in Gephi. Figure 15*a*–*f* is showing communication patterns between all users of #PanamaLeaks network for March, April, May, June, July and August, respectively. Figure 16 shows an overall visualization of the complete six months network from March 2017 to August 2017 using Yifan Hu algorithm. Our overall research methodology is shown in figure 6.

## 4. Results

In this section, we present series of experiments carried out in this study. We used social network analysis to address our research objective:

RO: Analysing the interaction patterns of central users to evaluate the influence in long-term online social media networks.

### 4.1. Conversation starter user

According to the two-step flow theory, information flows from mass media to opinion leaders and is then transmitted to the population via opinion leaders [23]. In the light of the above, we selected ‘siasatpk’ as our *conversation starter* as it is a mass media platform. As per the result of NodeXL, *conversation starter* topped the list of the most central users based on ‘betweenness centrality’. According to results of March, April, May, June and July ‘in-degree’ of ‘siasatpk’ is 211, 137, 203, 446 and 1463, respectively. Whereas, ‘out-degree’ of ‘siasatpk’ for March, April, May, June and July is 1, 3, 2, 7 and 22, respectively. ‘siasatpk’ who is playing the role as *conversation starters* in #PanamaLeaks network remains the first or second most influential participant of the network from March to July. The ‘in-degree’ of ‘siasatpk’ is zero in the month of August because ‘siasatpk’ stopped participating in discussion from August (table 1).

**Table 1.** Presenting ‘in-degree’, ‘out-degree’, ‘betweenness centrality’ and ‘rank’ of ‘siasatpk’ playing role as ‘Conversation Starter’ in the #PanamaLeaks network. The number ‘1’ in rank column presents that ‘Conversation Starter’ is the most central user in #PanamaLeaks network based on ‘betweenness centrality’.

month	in-degree	out-degree	betweenness	rank
March	211	1	492974.973	1
April	137	3	461067.857	2
May	203	2	975715.667	2
June	446	7	1219538.696	1
July	1463	22	7539507.557	1
August	0	0	—	—

**Figure 7.**
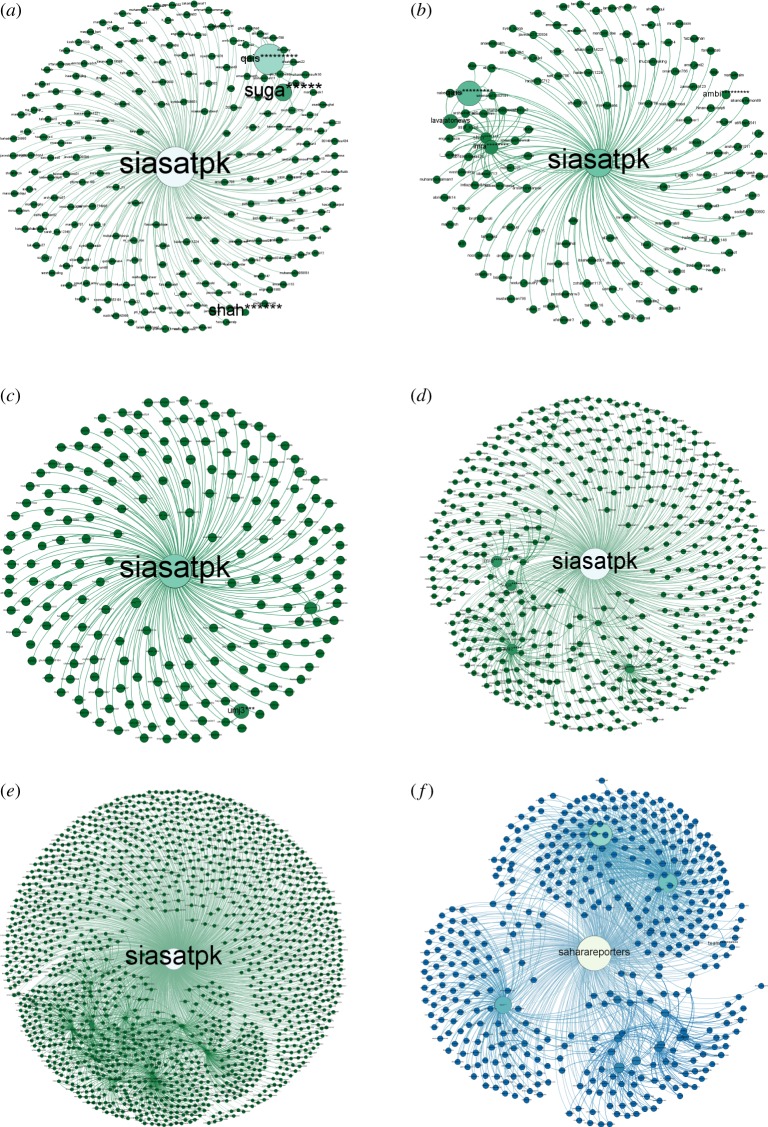
(*a*–*e*) Shows the connection pattern for ‘Conversation Starter’ of #PanamaLeaks network for the months of March, April, May, June and July, respectively. The middle node of every figure is ‘Conversation Starter’. The size of a node indicates its degree centrality. The colour of a node changes from green towards white with an increase in degree centrality. (*f*) Shows the connection pattern for ‘Conversation Starter’ of #NigeriaDecides network. The colour of a node changes from blue towards white with an increase in degree centrality. (*a*) ‘Conversation Starter’ of March, (*b*) ‘Conversation Starter’ of April, (*c*) ‘Conversation Starter’ of May, (*d*) ‘Conversation Starter’ of June, (*e*) ‘Conversation Starter’ of July and (*f*) ‘Conversation Starter’ of the #NigeriaDecides.

‘saharareporters’ with (in-degree = 345, out-degree = 13 and betweenness-centrality = 616332.423) is playing the role of *conversation starter* in the #NigeriaDecides case study, which we used for validation. Figure 7*a*–*e* shows the connection pattern of *conversation starter* for the months of March, April, May, June and July, respectively, of #PanamaLeaks network. Figure 7*f* shows the connection pattern of *conversation starter* of #NigeriaDecides network. Results of both case studies reveal the similar connection pattern for *conversation starter*. Mostly isolates are pointing towards the *conversation starter* (mentioning or retweeting tweets of *conversation starter*) in case study of #PanamaLeaks as well as #NigeriaDecides case study. In this context, *conversation starter* is acting as a hub in the online discussion networks.

### 4.2. Influencer user

There can be more than one *influencer* in the online discussion network, as result shows. Here we present top three *influencers* in #PanamaLeaks network of each month based on ‘betweenness centrality’. In March, ‘qais*********’ with (in-degree=139, (out-degree=12) is the third most central user of the network. The fourth most central user of the March network is ‘alir*********’ with (in-degree) = 150, out-degree = 1). ‘xaif************’ is the fifth most central user in March network with (in-degree = 90, out-degree = 1).

In April, ‘ptiofficial’ is the most central user of the network acting as an *influencer* in the network with (in-degree = 266, out-degree = 1) which means 266 other users have mentioned or retweeted ‘ptiofficial’ tweets. The third most central user is ‘qais*********’ with (in-degree = 107, out-degree = 12), and ‘amra*******’ with (in-degree = 116, out-degree = 2) is fourth most central user of April network.

In May, the most central user is ‘khaleejmag’ with (in-degree = 347, out-degree = 1). ‘diya********’ is the third most central user of the May network with (in-degree = 176, out-degree = 1) and ‘mary********’ with (in-degree = 59, out-degree = 0) is the fourth most central user of May network.

In June, second, fourth and sixth most central users are ‘dunyanews’, ‘imra********’ and ‘ptiofficial’ with (in-degree = 290, out-degree = 17), (in-degree = 105, out-degree = 0) and (in-degree = 80, out-degree = 1), respectively, playing role as *influencers* in the network.

In July, ‘imra********’ with (in-degree = 181, out-degree = 0), ‘dunyanews’ with (in-degree = 122, out-degree = 15) and ‘defencepk’ with (in-degree = 111, out-degree = 1) are the second, fourth and fifth most central user in the network.

In August, ‘shah******’ with (in-degree = 721, out-degree = 2) and ‘yada*********’ with (in-degree = 622, out-degree = 0) are the top two most central users in network.

The overall results of the top three *influencers* of every month are presented in table 2.

**Table 2.** Top three ‘Influencers’ of #PanamaLeaks network for every month based on ‘betweenness centrality’.

month	name of user	in-degree	out-degree	betweenness	rank
March	qais*********	139	12	315421.518	3
	alir*********	150	1	251914.020	4
	xaif************	90	1	200590.403	5
April	ptiofficial	266	1	596473.970	1
	qais*********	107	12	329445.157	3
	amra*******	116	2	288166.206	4
May	khaleejmag	347	1	1313558.254	1
	diya********	176	1	608312.772	3
	mary*********	59	0	551766.163	4
June	dunyanews	290	17	836335.327	2
	imra********	105	0	288851.658	4
	ptiofficial	80	1	213124.288	6
July	imra********	181	0	800178.225	2
	dunyanews	122	15	705731.185	4
	defencepk	111	1	601869.039	5
August	sheh********	721	2	328635.667	1
	yada********	622	0	194985.667	2

**Figure 8.**
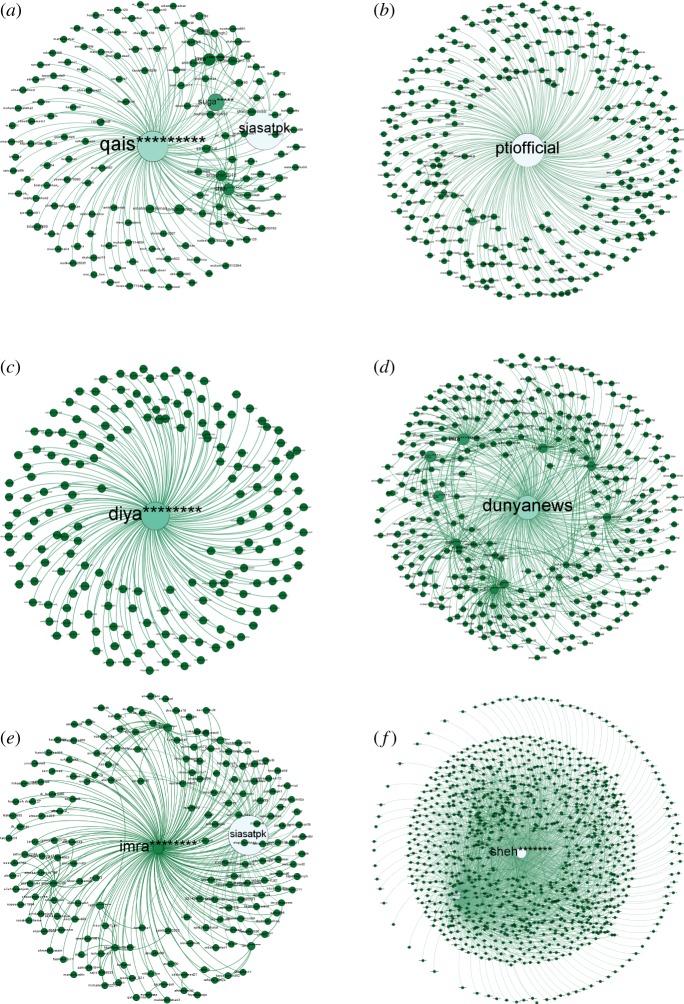
(*a*–*f*) Shows the connection pattern for ‘Influencer’ of #PanamaLeaks network for the months of March, April, May, June, July and August, respectively. The middle node of every figure is ‘Influencer’. The size of a node indicates its degree centrality. The colour of a node changes from green towards white with an increase in degree centrality. (*a*) Top ‘Influencer’ of March, (*b*) top ‘Influencer’ of April, (*c*) top ‘Influencer’ of May, (*d*) top ‘Influencer’ of June, (*e*) top ‘Influencer’ of July and (*f*) top ‘Influencer’ of August.

Figure 8*a* shows the connection pattern of ‘qais*********’ , who is playing a role as an *influencer* in the month of March. Figure 8*b* shows connection pattern ‘ptiofficial’, acting as the top *influencer* in the month of March. Many arrows pointing towards this node can be seen in figure 8*b* and most of these nodes are small presenting that mostly isolates like to mention or retweet *influencer* in the network. ‘khaleejmag’ is the top *influencer* of May network. Connection pattern of ‘khaleejmag’ is shown in figure 8*c*. June result shows, that *conversation starter* and *active engager* of June both mentioned top *influencer* of that month. However, most nodes pointing towards the ‘imra********’, the top *influencer* of June network are small, which means they are also isolates having low connectivity in the network. Figure 8*d*–*f* is showing the connection patterns of top *influencer* of June, July and August, respectively. All of the above figures are showing the same pattern, which shows connection pattern of *influencer* in the network remain similar over time.

**Figure 9.**
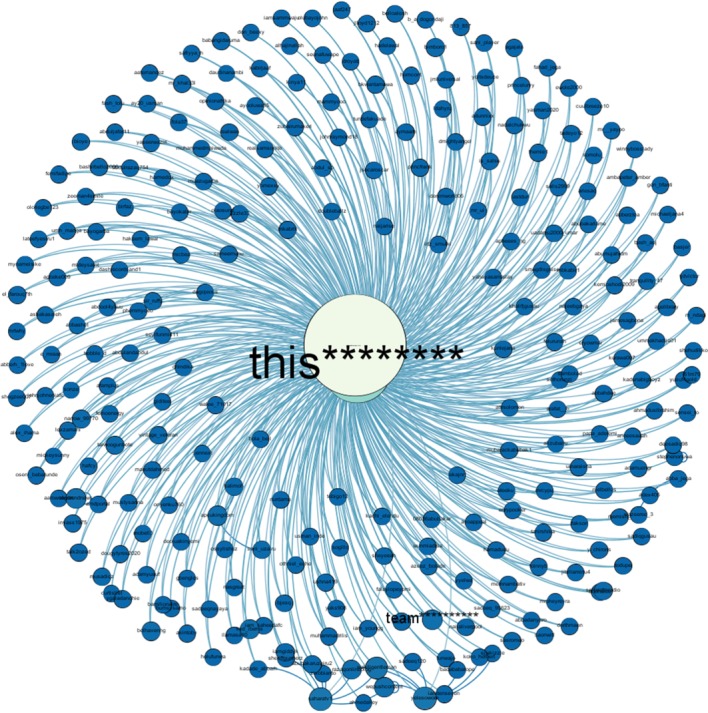
Top ‘Influencer’ of #NigeriaDecides case study. The node in the middle is the top ‘Influencer’ of #NigeriaDecides network. The size of a node indicates its degree centrality. The colour of a node changes from blue towards white with an increase in degree centrality.

In #NigeriaDecides network, “thisisbuhari”, “mbuhari”, “inecnigeria” are the top three influencers ranked by “betweenness centrality” playing the role as an influencer with (in-degree = 262; out-degree = 0; betweenness-centrality = 215550:136), (in-degree = 169; out-degree = 0; betweenness-centrality = 199496:055), (in-degree = 67; out-degree = 0; betweenness-centrality = 142580:817). Figure 9 shows the connection pattern of the top influencer “thisisbuhari” of #NigeriaDecides. The results of the both case studies #PanamaLeaks and #NigeriaDecides reveals the same connection pattern for the influencers in the online communication network.

### 4.3. Active engager user

The result shows that in the network of March, the second most central user with (in-degree = 0, out-degree = 65) is ‘suga*****’ an *active engager*. In network of April, ‘suga*****’ again acts as an *active engager* with (in-degree = 0, out-degree = 41) and became fifth most central user in the network. Seventh most central user ‘umj****’ is the *active engager* in network of May with (in-degree = 0, out-degree = 56) and in June it’s once again ‘suga*****’ with (in-degree = 0, out-degree = 96). In the network of July and August ‘lavajatonews’ with (in-degree = 0, out-degree = 65), (in-degree = 0, out-degree = 14), respectively, is playing the role as *active engager*. ‘lavajatonews’ is third most central user of July network and seventh most central user of August network playing role as *active engager* (table 3).

**Table 3.** Presenting ‘in-degree’, ‘out-degree’, ‘betweenness centrality’ and ‘betweenness-wise rank’ of the top ‘Active Engager’ of each month in the #PanamaLeaks network.

month	user name	in-degree	out-degree	betweenness	rank
March	suga*****	0	65	356338.123	2
April	suga*****	0	41	216878.111	5
May	umj3***	0	56	357661.301	7
June	suga*****	0	96	530891.138	3
July	lavajatonews	0	65	798249.408	3
August	lavajatonews	0	14	1360.000	7

**Figure 10.**
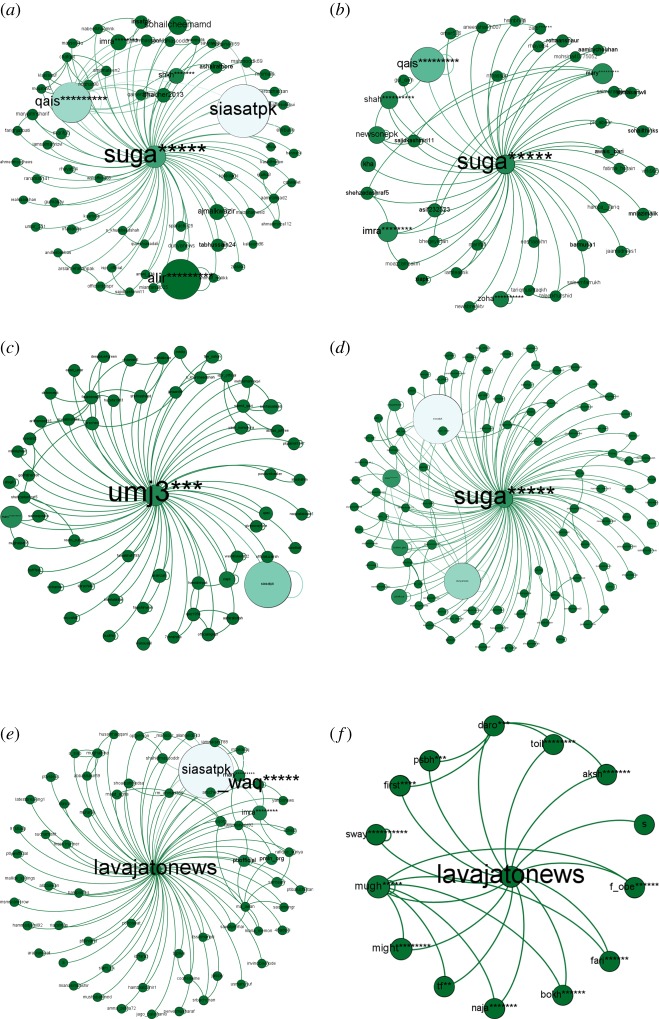
(*a*–*f*) Shows the connection pattern for ‘Active Engager’ of #PanamaLeaks network for the months of March, April, May, June, July and August, respectively. The middle node of every figure is ‘Active Engager’. The size of a node indicates its degree centrality. The colour of a node changes from green towards white with an increase in degree centrality. (*a*) Top ‘Active Engager’ of March, (*b*) top ‘Active Engager’ of April, (*c*) top ‘Active Engager’ of May, (*d*) top ‘Active Engager’ of June, (*e*) top ‘Active Engager’ of July and (*f*) top ‘Active Engager’ of August.

Figure 10 is showing connection pattern for *active engagers* from March to August of #PanamaLeaks network. In March, ‘suga*****’ is playing the role of *active engager* by retweeting/mentioning other participants of the network. ‘suga*****’ mentioned participant having high degree like ‘qais*********’, ‘alir*********’ and ‘siasatpk’; connection pattern of March *active engager* can be seen in figure 10*a*. ‘suga*****’ is again playing the role of *active engager* in April; figure 10*b* shows the connection pattern of ‘suga*****’. Mentioning ‘qais*********’, ‘mary********’ and ‘imra********’, three influencers in April network, is the reason for his central position in the network. ‘umj****’ plays the role as an *active engager* in May network. Figure 10*c* shows the connection pattern of ‘umj****’. By mentioning *conversation starter*, two *influencers* and other participants in June, ‘suga*****’ again is the *active engager*, as shown in figure 10*d*. Figure 10*e*,*f* shows the connection pattern for *active engager* of July and August month, respectively. Interestingly, ‘in-degree’ of all the above *active engagers* remain zero over the time. These results demonstrate that no other participants in the discussion have retweeted/mentioned *active engager* in their tweet from the start of the online discussion till the end.

**Figure 11.**
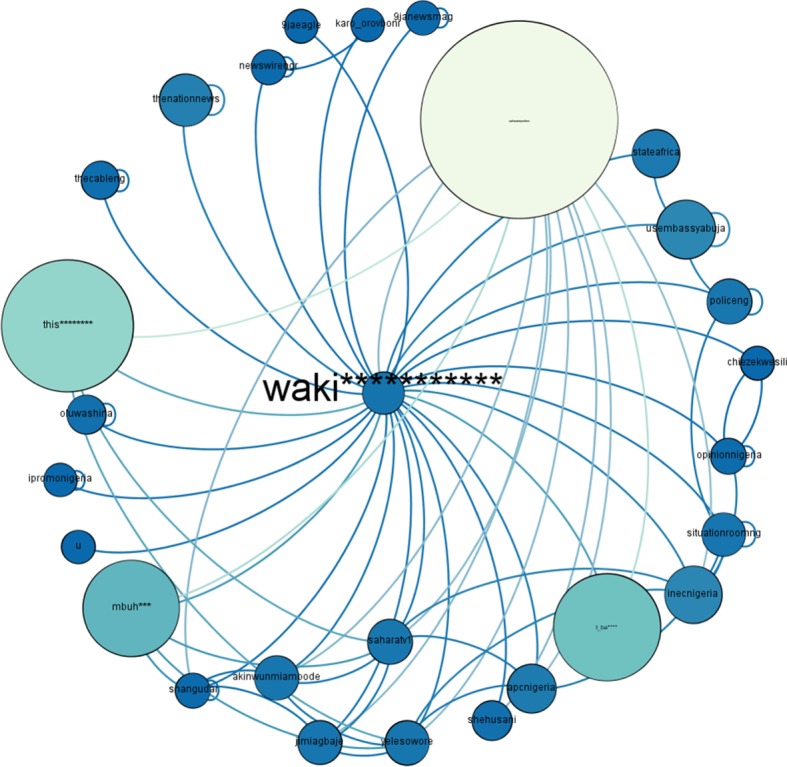
Connection pattern for ‘Active Engager’ of #NigeriaDecides network. The node in the middle of figure is ‘Active Engager’. The size of a node indicates its degree centrality. The colour of a node changes from blue towards white with an increase in degree centrality.

‘waki**********’ is playing role as an *active engager* in the network of #NigeriaDecides with (in-degree = 1 and out-degree = 26). He is the seventh most central user of the network. ‘Betweenness centrality’ of ‘waki**********’ is 112865.285. By mentioning/retweeting the *influencers* as well as the *conversation starter* in his tweets, he got this high ‘betweenness centrality’. Figure 11 shows the connection pattern of ‘waki**********’, results are consistent with the #PanamaLeaks case study.

### 4.4. Network builder user

‘shah******’ with only (in-degree = 0, out-degree = 2) is interestingly sixth most central user of the March network playing a role as *network builder*. With only (in-degree = 0, out-degree = 7) ‘ambitiousfree’ is the sixth most central user of April network because he mentioned two influencers ‘mary********’ and ‘amra*******’ in his tweets and built a strategic location in the network like ‘shah******’ in the network of March. ‘ali*******’ with (in-degree = 20, out-degree = 8) and ‘dezi*********’ with (in-degree = 0, out-degree = 18) are the *network builders* for month of May and June respectively. In July we could find ‘_waq*****’ as *network builder* on the thirteenth position with (in-degree = 5, out-degree = 21). In August with (in-degree = 0, out-degree = 3) ‘foro*******’ is the *network builder*. He is the fourth most central user in the network. No *influencer* in the network of all months has mentioned *network builders* in his/her tweet. However, by connecting two or more influential participants, *network builders* got central positions in the network. The overall results of the top *network builder* in the networks is shown in table 4.

**Table 4.** Presenting ‘in-degree’, ‘out-degree’, ‘betweenness centrality’ and ‘rank’ of top ‘Network Builder’ of each month in the #PanamaLeaks network based on ‘betweenness centrality’.

month	user name	in-degree	out-degree	betweenness	rank
March	shah******	0	2	186462.694	6
April	ambi********	0	7	162629.964	6
May	ali*****	20	8	295700.335	10
June	dezi********	0	18	267362.714	5
July	waq*****	5	21	276354.023	13
August	foro*******	0	3	2880.000	4

**Figure 12.**
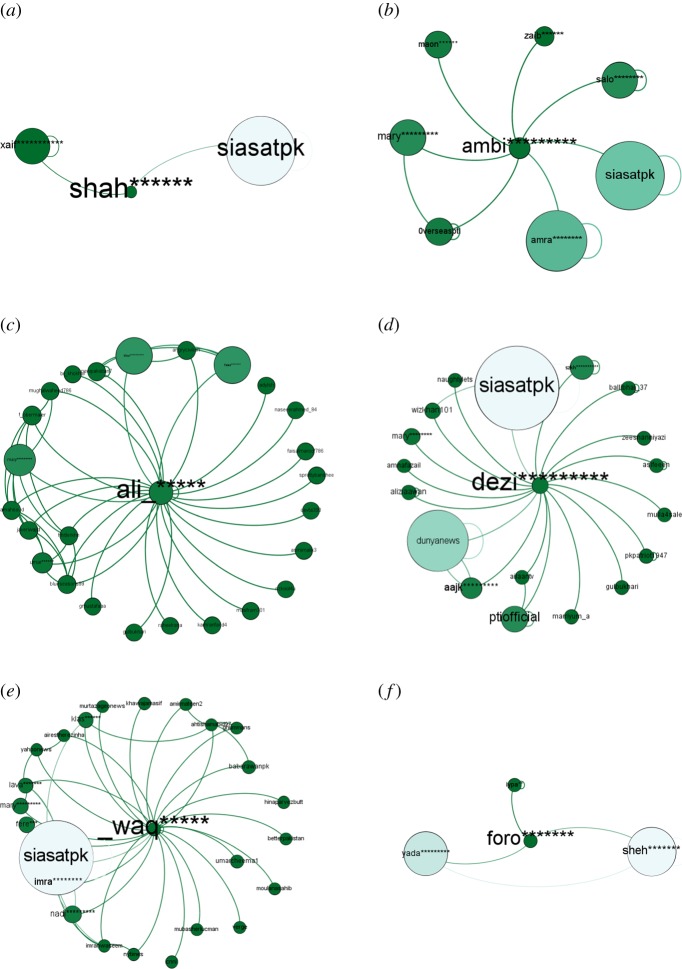
(*a*–*f*) Shows the connection pattern for ‘Network Builder’ of #PanamaLeaks network for the months of March, April, May, June, July and August, respectively. The middle node of every figure is ‘Network Builder’. The size of a node indicates its degree centrality. The colour of a node changes from green towards white with an increase in degree centrality. (*a*) Top ‘Network Builder’ of March, (*b*) top ‘Network Builder’ of April, (*c*) top ‘Network Builder’ of May, (*d*) top ‘Network Builder’ of June, (*e*) top ‘Network Builder’ of July and (*f*) top ‘Network Builder’ of August.

A node in the middle of figure 12*a* ‘shah******’ is *network builder* of March. With only two ‘out-degree’ links, he is the sixth most central user in the network. ‘shah******’ built a strategic location in the network by mentioning/retweeting only two participants having high ‘betweenness centrality’. Figure 12*b*–*f* shows the connection pattern for *network builder* of April, May, June, July and August, respectively. All the above-mentioned figures show a similar pattern of communication. All network builders have few ‘in-degree’ and ‘out-degree’ links; however, they build a strategic location in the #PanamaLeaks network by mentioning *influencers* or *conversation starter*.

**Figure 13.**
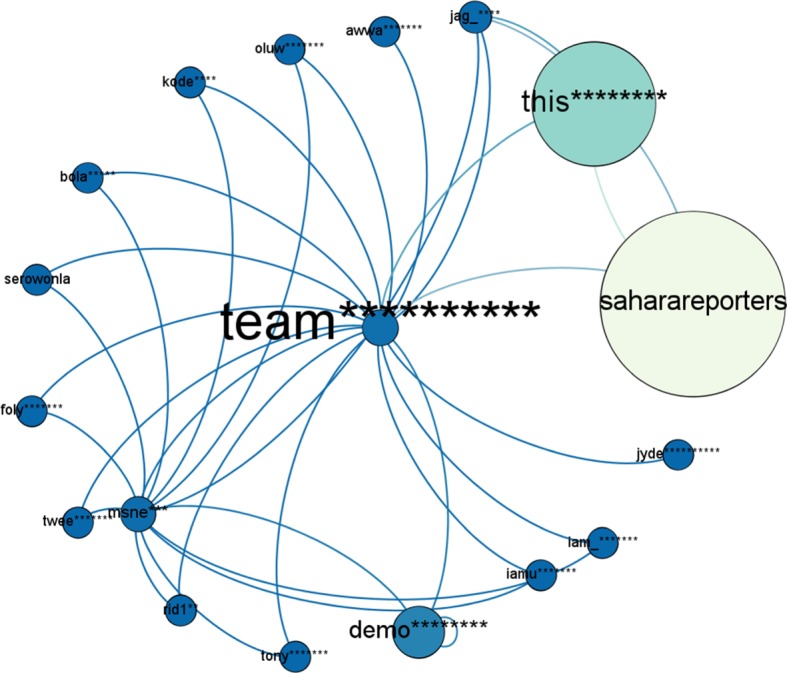
Connection pattern for ‘Network Builder’ of #NigeriaDecides network. The node in the middle is ‘Network Builder’. The size of a node indicates its degree centrality. The colour of a node changes from blue towards white with an increase in degree centrality.

‘team**********’ is playing the role as *network builder* in #NigeriaDecides network with (in-degree = 13 and out-degree = 3). He is the fifth most central user of #NigeriaDecides network by only mentioning two *influencers* ‘this********’, ‘demo********’ and *conversation starter* ‘saharareporters’ in the network. This result is also consistent with #PanamaLeaks network. Figure 13 is showing the connection pattern of the *network builder* of #NigeriaDecides network.

### 4.5. Information bridge user

The results reveal that there is no such user like the *information bridge* in #PanamaLeaks six months network. *Information bridge* is the user who mentions/retweets *influencer* tweet and that same tweet is then retweeted by *active engager* according to the study presented by Feng [30]. We could not identify any user with few ‘in-degree’ and ‘out-degree’ links, who has retweeted *influencer* tweet and then that same tweet is retweeted by *active engager*. We applied this approach to #NigeriaDecides case study [32] for validation purpose and according to the results; there exists no user like *information bridge*. Although, we were able to find *conversation starter*, *influencer*, *active engager* and *network builder* in #NigeriaDecides case study also.

### 4.6. Structural analysis

**Figure 14.**
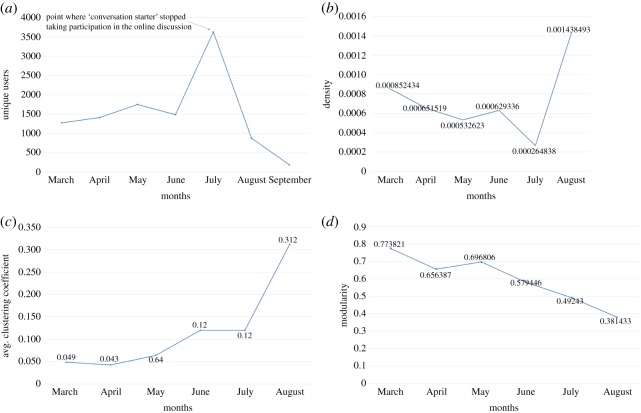
(*a*) Shows the number of unique users in the #PanamaLeaks network over the time. Overall density of #PanamaLeaks network is shown in (*b*). (*c*) Is showing average clustering coefficient of the network. (*d*) Is showing modularity of the network over the time.

Figure 14*a* shows the number of unique users participating in #PanamaLeaks online discussion every month. According to results, number of unique users participating in #PanamaLeaks discussion was 1277 in March. In April and May 1412 and 1750 unique users started participation in the online discussion, respectively. 1487 unique users joined the discussion in June and 3626 joined the discussion in July. However, there is a sudden decrease in a number of unique users participating in #PanamaLeaks discussion in August with 880 new unique users coming into the network. In September, there is a negligible number of users discussing the issue of Panama Leaks on Twitter. That is the reason we only analysed #PanamaLeaks network until August.

Figure 14*b* shows the density of #PanamaLeaks network over six months. A gradual decrease in the density of the network can be seen. However, there is a sudden increase in density after July. The average clustering coefficient of #PanamaLeaks from March to August is shown in figure 14*c*. The average clustering coefficient of the network starts from 0.049 in March and with gradual increase ends at 0.312 in August. Modularity of the #PanamaLeaks network is 0.773821, 0.656387, 0.696806, 0.579446, 0.49243 and 0.381433 for March, April, May, June, July and August, respectively (figure 14*d*).

**Figure 15.**
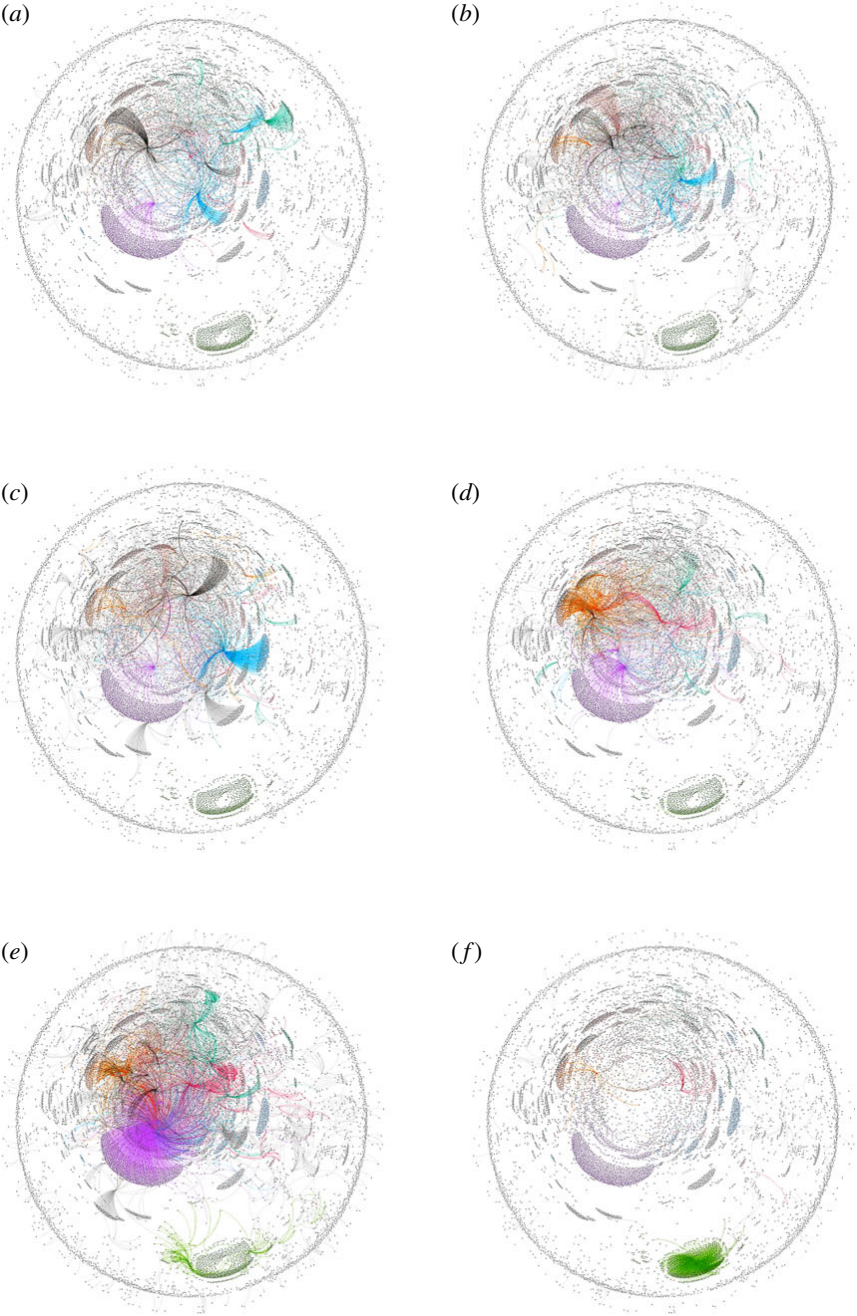
Dynamic graph visualization of #PanamaLeaks network for every month by Gephi using Yifan Hu algorithm. (*a*–*e*) Shows the #PanamaLeaks network for the month of March, April, May, June, July and August, respectively. Communities forming around ‘Conversation Starter’ and ‘Influencers’ are presented in different colours. (*a*) March, (*b*) April, (*c*) May, (*d*) June, (*e*) July and (*f*) August.

**Figure 16.**
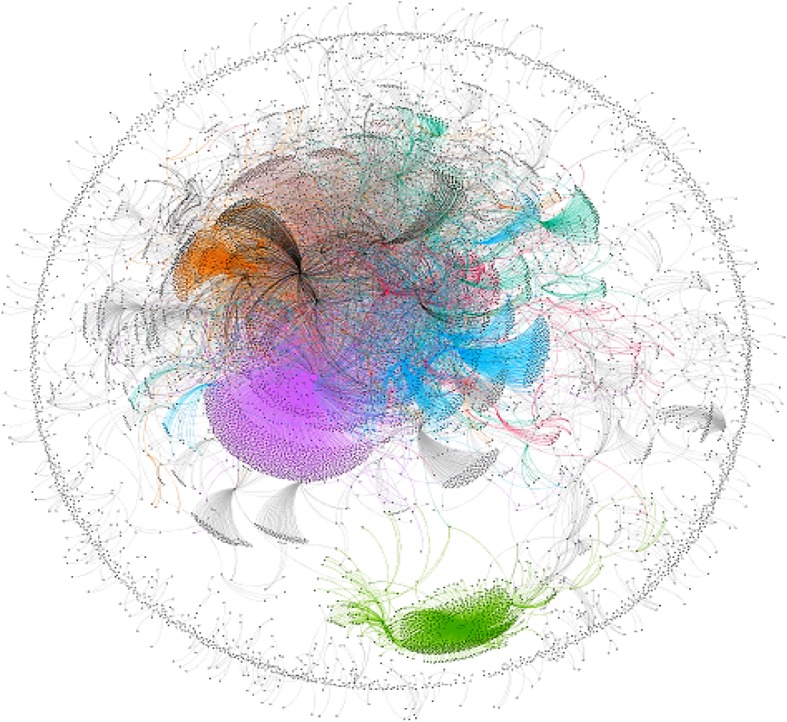
The combined network structure of #PanamaLeaks of six months.

Figure 15 shows interaction patterns of users participating in the #PanamaLeaks discussion. Many isolates are pointing towards the *influencers* and *conversation starter* in the network. Figure 15*a* shows the network of March and communities forming around *conversation starter* and *influencers* can be seen in the figure. These communities keep on growing until July, which can be seen in figure 15*b*–*f*. However, in August when major *influencers* and *conversation starter* left the discussion, communities started to dissolve which can be seen in figure 15*f*. A combined visualization of #PanamaLeaks network from March to August is shown in figure 16. A circle of participants around the edge of the graph can be seen. These participants are not connected to any *influencer* or *conversation starter* in the #PanamaLeaks network.

## 5. Discussion

In this study, we critically analyse the framework presented by Feng based on two-step flow theory of Kartz [30]. The results of this study reveal that there may not exist all of the central users identified by Feng in an online communication network. We have presented a detailed discussion about these central users in the following subsections. Additionally, we have also evaluated the roles of these central users in online discussion network. Moreover, this study illustrates how very few high-profile people are influencing the discussion in the online communication networks.

### 5.1. Conversation starter user

**Figure 17.**
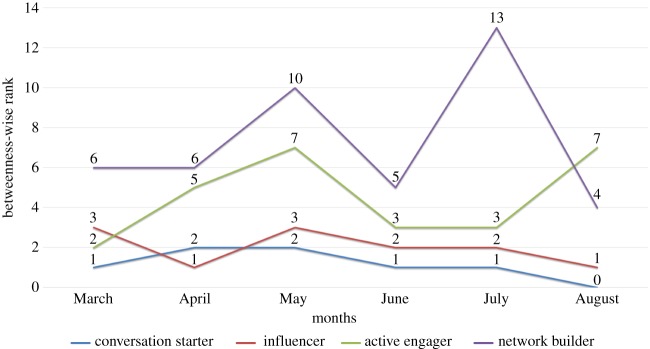
Changes in the ranking of central users from March 2017 to August 2017. Months are shown on the *x*-axis and betweenness-wise rank is shown along with the *y*-axis.

A *conversation starter* is one who is centre of the network and connects many ‘isolates’ who otherwise have few or no links in the network [30]. Results of this study reveal the same tendency, as many ‘isolates’ retweeted or mentioned ‘siasatpk’ in their tweets over the time. Some participants in the discussion with a high degree also mentioned *conversation starter* in their tweets but they are very few; mostly ‘isolates’ tend to connect with *conversation starter* to gain information. Since users with high connectivity are better at influencing information flow in the Twitter network [48]. This makes the *conversation starter* centre of the network by various incoming connections. However, the impact of the *conversation starter* on the online discussion can be evaluated by the number of users participating in online discussion. When *conversation starter* stopped participating in the trend (stopped tweeting about the topic), ‘isolates’ also lost interest in the online conversation. As results reveal that in August when *conversation starter* stopped participating in the online discussion, there is a sudden decrease in the number of new participants. However, while the *conversation starter* keeps on participating in the online discussion, results show that *conversation starter* remains on the top central positions in the network (figure 17).

### 5.2. Influencer user

**Figure 18.**
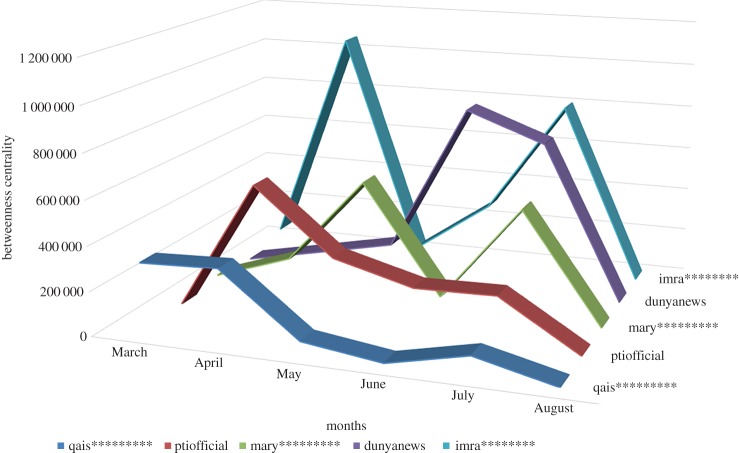
Changes in the ‘betweenness centrality’ of selected ‘Influencers’ in the #PanamaLeaks network over the time.

*Influencer* is a user in an online discussion network [30] with plentiful ‘in-degree’ and few ‘out-degree’ links. They are called opinion leaders in the network and isolates like to mention them in their tweets. Isolates also like to retweet opinion leader’s tweet and these opinion leaders are usually mass media organizations or celebrities in different fields of life [30]. Opinion leaders create frequent content to influence the opinion of other participants in the online discussion. Information is going from opinion leaders to users with less connectivity and this supports the argument by Dahlberg [33] and Murdock & Golding [34] that opinion leaders by using their network connectivity replicate asymmetric offline power in the online discussion. The participant like *influencer* who has high degree act as a hub in the network [49]. However, opinion leaders’ influence is not consistent in the online communication network as the results show in #PanamaLeaks case study, the power of an *influencer* to influence other participants in the online communication network can increase/decrease over time. Figure 18 shows the changes in ‘betweenness centrality’ of five selected *influencers* in the #PanamaLeaks network over six months.

### 5.3. Active engager user

A user with many ‘out-degree’ and none or few ‘in-degree’ links is an *active engager* in the network [30]. Results reveal that there always exists an *active engager* in an online communication network over time. However, the position of *active engager* is not consistent and another *active engager* can take his/her place with the passage of time. *Active engager* is also among the top 10 most central users of the network. Results show that *active engager* is one who is active with the intention to get information during an online discussion. *Active engager* may not become an opinion leader as no participant in the online discussion has mentioned him in his/her tweet over six months period.

### 5.4. Network builder user

*Network builder* in a network has few or none ‘in-degree’ and few ‘out-degree’ links [30]. His primary role in the network is to associate two or more influential nodes in the network. By this, *network builder* builds a strategic location in the online communication network and gets high centrality. However, in [30] ‘wnkr’ is a *network builder* and its in-degree is ‘0’. Which means no participant in the discussion either has mentioned or retweeted ‘wnkr’ in their tweets. In #PanamaLeaks network, *network builders* follow the same tendency. In this context, we can conclude that *network builder* does help in creating a connection between two or more *influencers* in the online discussion network. However, *influencers* usually do not mention or reply to their tweets. Therefore, their impact on the opinion formation in an online discussion network is petite.

### 5.5. Information bridge user

*Information bridge* is a user with few ‘in-degree’ and ‘out-degree’ links, who connects an *influencer* and *active engager* as noted in [30]. However, results of the longitudinal network of this study show that there is no such user like *information bridge* existing in the online discussion network. Furthermore, for validation of our results we used another published case study #NigeriaDecides [32]. The result shows that in the case study #NigeriaDecides, there does not exist any user like *information bridge* who has retweeted/mentioned *influencer* in his/her tweet and then that tweet is retweeted by *active engager*. *Active engager’s* primary role in the network is to engage with other users [30], and for this purpose; *active engager* likes to retweet mostly *influencer/conversation starter* tweets. *Active engagers* do not usually retweet/mention ‘isolates’ in their tweets according to the six months network of #PanamaLeaks network. In this context, we can conclude that there is an insignificant chance that an *active engager* retweets the same tweet in which a person with few ‘in-degree’ and ‘out-degree’ has mentioned *influencer*.

### 5.6. Network structure

The results of this study show that unique users increase until June and then there is a sudden decrease in the number of unique users as shown in figure 14*a*. When *conversation starter* stopped participating in the online discussion there is a sudden decrease in the number of new participants. This shows the effect of *conversation starter* on the online discussion as users with high connectivity can influence the participation of users [50]. Most of the unique users in the online discussion connected with *conversation starter* and *influencers* are participants with low degree. In this context, opinion leaders have the power to keep isolates interested in the online discussion. Network density can decrease with the number of users increasing in the network and vice versa [21], the density of the network is showing the same tendency.

The clustering coefficient of *conversation starter* and top *influencer* of March, April, May, June and July is 0.00, 0.002, 0.00, 0.001, 0.00 and 0.004, 0.00, 0.00, 0.005, 0.005, 0.002, respectively. Results show that *conversation starter* and top *influencer* of every month have mostly zero clustering coefficient over the time. Presenting that almost all the participants connected with *conversation starter* and *influencers* have no interest in sharing information with each other in the online discussion network. However, all the participants connected with these opinion leaders are there to get information as these highly connected participants in the online social network are a source of information. Reciprocated vertex pair ratio of *conversation starter* and *influencers* in the network remains zero over the time supporting our above argument that these opinion leaders are there to diffuse information to isolates without any intention of getting information from the less-connected participants participating.

Highly dense connections between the nodes of a community and sparse connection between different communities show high modularity in a social network [51]. Results show the same tendency, as there exist dense connections between the participants following the same opinion leader and sparse connections between the followers of different opinion leader in the online discussion network. Communities formed around the *conversation starter* and *influencer* are closely connected and these closely connected communities can help in fast information diffusion in the network [52], as strong modularity in a social network enhances information diffusion within a community and obstructs it in inter-community [53]. The role of *conversation starter* and *influencer* in an online discussion network can be seen by this argument as well. In the presence of both *conversation starter* and *influencer*, information diffusion within a community is strong due to high modularity. However, the links between the different communities are very few, showing weak ties between the communities of online discussion. Participants of the online discussion tend to cluster around the influential participants and with the passage of time in the presence of *conversation starter* and *influencers*, participants became less independent as modularity decreased [54].

The role of *network builder* and *active engager* in the overall structure of the online communication network is connecting two or more otherwise sparse communities. Even few weak ties in the online social network can help in diffusing information within the different communities [55]. *Network builder* and *active engager* are doing the same, connecting mostly the communities formed around *conversation starter* and one or more top *influencers* in the network. On all the above arguments, we can conclude that *conversation starter* and *influencers* are the information source in the network. *Network builder* and *active engager* are working as the bridge for diffusing this information between the different communities of society. However, the power to control and diffuse information did not remain the same over time as par result of the #PanamaLeaks network. Moreover, with many participants having low degree pointing towards the *conversation starter* and *influencer*, we can conclude that they play a role as a hub in the online discussion network.

As far as the discussion regarding #PanamaLeaks online communication network, we can see how these participants are playing a central role. From the start of the case in Supreme Court of Pakistan until the final verdict, *conversation starter* and *influencers* are influencing the overall discussion about #PanamaLeaks by creating tweets and using their social network connectivity. Results show that they are successful in diffusing the information as well as getting the attention of participants during the whole discussion. Our findings also demonstrated that these participants are mostly isolates with the intention to gain information about the #PanamaLeaks discussion, resulting in creating communities around the *conversation starter* and *influencers*. We can also see the role of *network builder* and *active engager* in the #PanamaLeaks online discussion as they have acted as the bridge in connecting the communities formed around political leaders and media of Pakistan. However, in the online discussion about Panama Papers in Pakistan, we can see that highly influential participants are very few compare to isolates. In light of the above arguments, we can conclude that even in this era of digital communication where everyone is equipped with the power to express their opinions to others in online discussion platforms. Still very few individuals by replicating their offline asymmetric power in online platforms can influence opinions [33,34].

## 6. Conclusion

In this study, we investigated the variations in the power of opinion leaders in online communication networks. We critically analysed the framework presented by Feng in short-term and long-term manner. We adopted #PanamaLeaks as a case study. Our finding demonstrated that there might not exist all of the central users identified by Feng in an online communication network. We validated our results by another published case study of #NigeriaDecides. Furthermore, our study contributed by identifying the impact of these central users over the time. Our findings can help to predict the connection patterns and how an online communication network can transform in the future. Moreover, for initializing an online marketing campaign, targeting these central users can make a great impact on the information diffusion in the online social networking platforms.

Future studies based on this framework might include the content of the tweets to better understand the relationship between the participants of the online communication network. A signed graph can be constructed to analyse communities having positive and negative opinion about the trend. That can help to find the communication patterns among communities of the online communication network. Moreover, we suggest that future studies add more variables to this model as we have only focused on social network analysis measures based on centrality.

## Data accessibility

This article does not contain any additional data.
